# A provider survey of cystic fibrosis related diabetes screening and management practices at North American CF centers

**DOI:** 10.3389/fendo.2023.1183288

**Published:** 2023-05-18

**Authors:** Rebecca Hicks, Katie Larson Ode, Tim Vigers, Christine L. Chan

**Affiliations:** ^1^ University of California, Los Angeles, Division of Pediatric Endocrinology, Los Angeles, CA, United States; ^2^ Department of Pediatrics, University of Iowa, Iowa City, IA, United States; ^3^ Fraternal Order of Eagles Diabetes Research Center, University of Iowa, Iowa City, IA, United States; ^4^ Department of Pediatrics, University of Colorado Anschutz Medical Campus, Aurora, CO, United States

**Keywords:** cystic fibrosis, diabetes screening, CFRD, oral glucose tolerance test, glycemia, continuous glucose monitor

## Abstract

**Background:**

Cystic Fibrosis Foundation (CFF) Guidelines recommend annual screening for cystic fibrosis related diabetes (CFRD) with an oral glucose tolerance test (OGTT). However, screening rates remain consistently low. We conducted surveys of 1) US CF center directors and 2) Endocrinologists affiliated with the CFF-sponsored EnVision program to characterize CFRD screening practices, describe provider perceived barriers to screening, and identify strategies for improving screening.

**Methods:**

The surveys queried OGTT protocols, alternate screening strategies, and perceived barriers to screening. CF center characteristics and procedures for coordinating OGTTs were compared between centers achieving ≥50% versus <50% OGTT completion. Endocrinologists received additional questions regarding OGTT interpretation and management.

**Results:**

The survey response rate was 18% (51/290) from CF Centers and 63% (25/40) from Endocrinologists. The majority (57%) of CF centers utilized 2 OGTT timepoints (0,120 min). The majority (72%) of Endocrinologists utilized 3 timepoints (0,60,120 min). Four percent of CF centers and 8% of Endocrinologists utilized other timepoints. Forty-nine percent of CF centers reported ≥50% OGTT completion in the past year. Completion of ≥50% OGTT was 5 times more likely when patient reminders were consistently provided (p = 0.017). Both CF Centers and Endocrinologists employed alternative screening strategies including HbA1c (64%, 92%), fasting plasma glucose (49%, 67%), continuous glucose monitoring (30%, 58%), and home fingerstick monitoring (55%, 50%).

**Discussion:**

OGTT is the gold standard screening method for CFRD, but completion rates remain suboptimal, practice variation exists, and many providers utilize alternate screening strategies. Systematic reminders may improve completion rates. Studies to improve our approach to CFRD screening are urgently needed.

## 1 Introduction

Cystic fibrosis related diabetes (CFRD) is prevalent in 2% of children <10 years, 19% of adolescents, and up to 50% of adults with cystic fibrosis (CF) (1). CFRD has unique significance in the CF population as it is associated with declining pulmonary function, increased frequency of pulmonary exacerbations, worse nutritional status, three-fold increased risk of mortality (1, 2), and diabetes-related microvascular complications (3). Proactive surveillance is important as the onset is insidious, and early diagnosis and optimization of glycemic control is associated with improved pulmonary function and nutrition, decreased frequency of pulmonary exacerbations, and decreased overall mortality (1–5). The CF Foundation (CFF) recommends 2-hour oral glucose tolerance testing (OGTT) with 0 minute (fasting glucose) and 120 minutes (post-dextrose containing beverage) time points as the gold standard screening method for CF patients ≥10 years of age (4), yet CFF patient registry data have consistently demonstrated low OGTT screening rates (≤30% of adults and ≤60% of youth nationally) (2, 6).

Although US CFF CFRD screening guidelines are unchanged since 2010, US screening rates have remained concerningly low, and substantial knowledge gaps persist regarding how to improve screening. One such gap is a lack of data on provider perceived barriers to CFRD screening. Therefore, our aims were to conduct a survey of 1) CF center directors in the US and 2) Endocrinologists affiliated with the EnVision program (a CFF program to foster Endocrinologists in the care of CF related endocrinopathies) in order to 1) better characterize center-specific CFRD screening practices, 2) describe provider perceived barriers to screening, and 3) identify potential strategies for improving CFRD screening.

## 2 Materials and methods

### 2.1 Survey development and distribution

An advisory committee of Pediatric Endocrinologists with expertise in CF (authors RH, KLO, and CC) guided the development of two REDCap surveys, which were further revised based on feedback from two additional Pediatric Endocrinologists and a Pediatric Pulmonologist/CF center director. Each survey consisted of 23 questions pertaining to CFRD screening practices (**Supplementary Table 1**). The CF center survey included questions about CF center practice settings, patient demographics, details of OGTT protocols, OGTT sampling time points, whether glucose monitoring is recommended during or after gastrostomy (G-tube) feeds, use of alternate diabetes screening strategies, and provider perceived barriers. The Endocrinologist survey was similar, however it excluded questions regarding OGTT scheduling processes, and it included additional questions regarding interpretation of OGTT results and management approaches to abnormal OGTTs and other measures of glycemia.

Respondents for both surveys were asked to rank their top 5 perceived barriers to OGTT screening in order from greatest to least. Additionally, optional free text comments were solicited regarding approaches to overcoming barriers to OGTT screening, as well as general comments related to CFRD screening.

The link to Survey #1 was sent out *via* email distribution to CF Center Directors (n=290). The Center Directors were provided the option to designate an alternate individual to complete the survey, at their discretion. The link to Survey #2 was sent out *via* group email distribution to Endocrinologists within the EnVision I and II programs (n=40). The surveys were open from December 2021- June 2022. The survey was approved by the Institutional Review Board at the University of California Los Angeles, and informed consent was obtained from survey participants prior to the first survey question.

### 2.2 Analysis plan

Survey responses were summarized using frequencies with percentages for each categorical variable. CF centers were categorized into those reporting ≥50% versus <50% completion rates in the preceding year. We hypothesized that the following variables would be positively associated with ≥50% completion rates: <50% of patients with state funded public health insurance, an identified Endocrinologist, a standardized process for ordering OGTTs, an identified team member for ordering OGTTs, an identified team member for providing patients with instructions for completing the OGTT, performing the OGTT in clinic, and consistently providing patient reminders for the OGTT.

Strategies for OGTT screening and frequency of alternate screening strategies were compared using chi-squared or Fisher’s exact tests. Data were summarized and analyzed in R (version 4.2.2).

Themes from comments regarding approaches to overcoming OGTT barriers as well as overall perceptions on CFRD screening were summarized.

## 3 Results

### 3.1 Survey response rates

The survey response rate for CF Centers was 18% (51/290), and the survey response rate for Endocrinologists was 63% (25/40).

### 3.2 CF center and endocrinologist characteristics

#### 3.2.1 CF center characteristics

Forty-four percent of survey responses were from the CF Center Director, and the remainder from designees (**Table 1A**). Eighty percent are affiliated with an academic institution. The majority of centers have between 25 - <75% of patients with public health insurance, with a fairly even split between pediatric and adult centers (both approximately 40%); 18% have a mixed pediatric and adult patient population. The vast majority (92%) reported having an identified Endocrinologist for referrals, with nearly half having an embedded Endocrinologist in a coordinated multidisciplinary CF clinic.

**Table 1A:** Characteristics of CF Centers Responding to Survey.

Number of Survey Responses	**Total Number of Survey Responses**	**Number of Unique CF Centers in Survey Responses**	Unique Survey Respondents^*^	**Complete Surveys**	**Survey Response Rate^#^**
	59	51	54/59 (92%)	47/59 (80%)	51/290 (18%)
Role	**CF Center Director**	**CF Center Coordinator**	**CF Pulmonologist**	**Other**	** **
	24/54 (44%)	18/54 (33%)	8/54 (15%)	13/54 (7%)	
Setting	**Center Affiliated with Academic Institution**	**Community-Based Clinic/Center without Academic Affiliation**	**Other**		
	39/49 (80%)	7/49 (14%)	3/49 (6%)		
Percent of Patients with State-Funded Health Insurance	**<25%**	**25 - <50%**	**50 - <75%**	**>= 75%**	**I don't know**
	4/48 (8%)	20/48 (42%)	17/48 (35%)	3/48 (6%)	4/48 (8%)
Patient Age Group	**Pediatric**	**Adult**	**Both Adult and Pediatric**	** **	** **
	21/49 (43%)	19/49 (39%)	9/49 (18%)		
Identified Endocrinologist for CF Center	**Yes**	**No**	**Other**	** **	** **
	44/48 (92%)	1/48 (2%)	3/48 (6%)		
Identified Endocrinologist in Coordinated Multidisciplinary Clinic	**Yes**	**No**			
	22/48 (46%)	26/48 (54%)			

^*^3 CF Centers had more than 2 unique survey respondents (same CF Center, different Role).

^#^% of Survey Responses from Unique CF Centers Compared to Total Accredited and Affiliate CF Centers.

#### 3.2.2 Endocrinologist characteristics and their practice settings

Fifty-six percent of Endocrinologists completing the survey had Pediatric training, 32% Adult, and 12% had training in combined Adult and Pediatric Endocrinology (**Table 1B**). The vast majority (92%) practice at an academic institution. Forty percent see patients in a multidisciplinary CF clinic AND patients are referred to their Endocrine and/or Diabetes clinic, while 32% only see patients in a multidisciplinary CF clinic, and 20% only see patients referred to their Endocrine and/or Diabetes clinic.

**Table 1B:** Characteristics of Endocrinologists Responding to Survey.

Endocrinologist Background^*^	**Pediatric**	**Adult**	**Medicine-Pediatrics (Adult and Pediatrics)**	
	14/25 (56%)	8/25 (32%)	3/25 (12%)	
Age Group(s) of Patients	**0 - 21 yr**	**0 - 25 yr**	**18 - >25 yr**	**All Age Groups**
	5/25 (20%)	6/25 (24%)	8/25 (32%)	6/25 (24%)
Endocrine practice description	**Patients referred to Endocrine and/or Diabetes clinic**	**See patients in multidisciplinary CF center**	**Patients referred to Endocrine and/or Diabetes clinic AND see patients in multidisciplinary CF center**	**Other**
	5/25 (20%)	8/25 (32%)	10/25 (40%)	2/25 (8%)
Setting	**Center Affiliated with Academic Institution**	**Community-Based Clinic/Center without Academic Affiliation**	**Other**	
	23/25 (92%)	2/25 (8%)		

*Survey Invitation was Emailed to 40 Adult and Pediatric Endocrinologists in the EnVision I and II cohorts.

### 3.3 Alternative glucose sources for OGTT testing

The glucose beverage used for OGTT testing is often unpalatable and can present a barrier to OGTT testing. Therefore, authors have advocated for alternative glucose sources (i.e., juice, pop/soda, jelly beans, etc.) (7). When providers were surveyed regarding the use of alternative glucose sources for OGTT testing, CF centers reported frequencies of: sometimes 4% (2/47), rarely 21% (10/47), never 70% (33/47), and I don’t know 4% (2/47). Endocrinologists’ responses were as follows: sometimes 8% (2/25), rarely 20% (5/25), and never 72% (18/25).

### 3.4 CF center characteristics associated with ≥50% OGTT completion rates

Forty-nine percent of all CF centers reported ≥50% OGTT completion in the past year (**Table 2**). Notably, pediatric CF centers had the highest ≥50% OGTT completion rates (15/21 total, including 1 response omitted = 75%), followed by combined adult and pediatric centers (4/9 = 44%), then adult centers (4/19 total, including 2 responses omitted = 24%). This difference was statistically significant (p = 0.007). When screening protocols were compared between centers achieving ≥50% vs <50% OGTT completion, the only statistically significant difference was centers that always or almost always provide reminders to patients are about 5 times more likely to have OGTT completion rates ≥50% (p = 0.017). Reminder methods cited included: phone calls, communication *via* electronic medical record system, and emails; 22% (9/41) of centers who reported giving reminders commented that the reminders occurred at a clinic visit.

**Table 2:** Characteristics of CF Centers Reporting >50% OGTT Completion Rates.

Association of CF Center Characteristics with Report of >50% OGTT Completion Rates^#^				p value
Patient Age Group(s) of Center	Adult	Both Adult and Pediatric	Pediatric	
	4/19 (24%)^¥^	4/9 (44%)	15/21 (75%)	0.007^*^
Health Insurance	More Than 50% State Funded	Less Than 50% State Funded		
	9/22 (45%)	11/21 (55%)		0.639
Identified Endocrinologist	Yes	No		
	21/42 (50%)	2/4 (50%)		1

Association of OGTT Process with Report of >50% OGTT Completion Rates^#^				p value
Protocol or Standardized Method for Ordering OGTT	Yes	No		Not Applicable^^^
	23/44 (52%)	0 (0%)		
Identified Role for Placing OGTT Orders	1 Individual in Role	2 Individuals in Role	3 or More Individuals in Role	
	11/24 (46%)	7/14 (50%)	5/8 (50%)	0.683
Identified Role for Providing Patient Directions for OGTT	1 Individual in Role	2 Individuals in Role	3 or More Individuals in Role	
	8/20 (40%)	9/12 (75%)	6/14 (43%)	0.374
Perform OGTT in clinic	Yes	No		
	12/20 (60%)	11/26 (42%)		0.639
Always/Almost Always Provide OGTT Reminders	Yes	No		
	20/31 (65%)	3/13 (23%)		0.017^*^

^#^For Each Specific Categorical Variable Response: Numerator = number of CF Centers reporting >50% OGTT Completion Rate with specific categorical response; Denominator = Total number of CF Centers respondents with specific categorical variable response.

^¥^2 Adult centers and 1 Pediatric center responding to survey did not specify OGTT completion rates.

^^^44/46 centers (95.7%) have a protocol or standardized method for ordering OGTTs. Unable to perform a statistical test because there were 0 centers without a protocol and over 50% OGTT completion.

^*^statistically significant (p<0.05).

### 3.5 Pediatric, adult, and combined CF center OGTT process characteristics

OGTT ordering and coordination processes were also compared among pediatric vs adult vs combined CF centers. Pediatric and combined centers were more likely than adult centers to see patients with state funded health insurance (62%, 57%, vs 19%, respectively, p = 0.027). Pediatric and combined centers were also more likely than adult centers to have OGTTs completed in clinic (57%, 56%, vs 16% respectively, p=0.016). Patient reminders for scheduling the OGTT were not statistically different when comparing pediatric, adult, and combined CF center responses (p = 0.446).

### 3.6 CFRD screening practices

#### 3.6.1 OGTT time points

Current CFF CFRD guidelines recommend venous glucose to be sampled at 0 and 120 min only (4). However, additional timepoints are often utilized (8). Fifty-seven percent of CF centers surveyed obtain venous glucose levels at 0 and 120 min only, 38% at 0, 60, and 120 min, and 4% obtain additional intermediate timed venous samples. Of the Endocrinologists surveyed, 20% reported glucose levels obtained at 0 and 120 min at their center, 72% reported 0, 60, and 120 min, and 8% reported obtaining additional intermediate glucose time points.

#### 3.6.2 Screening for people with CF on gastrostomy feeds

For individuals ≥10 years old starting G-tube feeds, current CFF CFRD screening guidelines recommend glucose monitoring during or after feeds (4), however adherence to this recommendation is unclear from CF registry data (6). In our survey, CF centers reported frequency of recommended glucose monitoring after G-tube feeding initiation as follows: always or usually 15% (7/47), sometimes 30% (14/47), rarely 40% (19/47), and never 15% (7/47).

#### 3.6.3 Alternative screening strategies:

Although current CFF guidelines do not recommend alternatives to OGTT testing, use of alternative strategies is reported in the literature (9–22). Both CF Centers and Endocrinologists surveyed reported using alternate strategies to evaluate for diabetes such as Hemoglobin A1c (HbA1c) (64%, 92%), fasting plasma glucose (49%, 67%), continuous glucose monitoring (CGM) (30%, 58%), and fingerstick glucose monitoring (55%, 50%), respectively.

### 3.7 Barriers to OGTT screening

The following barriers to OGTT screening were cited by CF Center Directors: 91% (43/47) selected duration of the test, with 47% of this group (20/43) ranking it the overall greatest barrier. The need for fasting before the test was the second most cited barrier by 72% (34/47) and reported as the greatest barrier by 18% of this group (6/34). The need to have the test performed in the morning was selected by 66% (31/47) and ranked as the greatest barrier by 19% (6/31). Other barriers selected by some as the greatest barrier (albeit at lower rates) included: patient doesn’t think OGTT is important/necessary (5/33 = 15%), patient doesn’t like the taste of Glucola or does not tolerate it (2/26 = 8%), and number of blood draws (2/26 = 8%).

Endocrinologists reported the most significant barrier to be the duration of the test (5/18 who selected this as a barrier felt it was the greatest barrier) and “Patient doesn’t like the taste of or doesn’t tolerate Glucola” (5/12 selecting this considered it the greatest barrier). The most frequently reported overall barrier (reported by 90% (19/21) of Endocrinologists) was “patient doesn’t think OGTT is important/necessary,” although only 16% (3/19) citing it ranked it as the greatest barrier to completion of testing. The need for fasting before the test was reported by 71% (15/21), with 20% (3/15) ranking it as the greatest barrier. The need for the test to be performed in the morning was also ranked by 71% (15/21) of respondents, with 13% (2/15) ranking it the greatest barrier. The number of blood draws needed was ranked by 57% (12/21) of Endocrinologists as a barrier, however it was not ranked as the greatest barrier by any. In fact, it was ranked as the lowest barrier by 46% (6/13).

Themes from free text comments from both surveys regarding barriers overlapped, and included limitations imposed by laboratories (i.e., appointment availability; minimum patient age and weight requirements for calculation of the Glucola dose); the need for a separate appointment apart from CF clinic visits; the distance of the OGTT testing location from the patient’s home and/or CF center; patients forgetting to fast or intentionally not fasting because they feel the test is too long or unnecessary, as well as patient anxiety from traumatic past experiences with the test (i.e., previous episode of vomiting, fainting, and/or hypoglycemia). Other themes included provider resistance due to perception that the OGTT may not be needed in pancreatic sufficient individuals who are clinically stable with a normal HbA1c, especially with highly effective modulator therapy (HEMT).

### 3.8 Overcoming barriers to OGTT screening

Themes from free text comments regarding approaches to overcoming barriers to OGTT screening included the following:

- Long-term Quality Improvement (QI) projects (increased OGTT rates only while QI project is active)
- Increasing flexibility in timing and locations of the OGTT (i.e., ability to perform these during clinic – ideally with a designated quiet area rather than in the main waiting area, in infusion centers, locations near a patient’s home, morning and afternoon times, during school breaks, use of in-home phlebotomy)
- Placing an IV to avoid more than one venipuncture
- Early (prior to 10 years old) and ongoing education to patients and CF team members regarding importance of CFRD screening *via* OGTTs
- Standardizing/streamlining the process (i.e., educating schedulers, collaboration amongst team members)
- Extending hospital stays to obtain an OGTT
- Utilizing a standardized glucose beverage with a different flavor or from a different manufacturer
- Prescribing ondansetron prior to drinking Glucola to decrease nausea
- Utilizing multiple types of reminder methods by the same center
- Utilizing incentives (i.e., gift card raffle for routine patient care, monetary compensation when done for a research study).

### 3.9 Endocrinologists’ practice management based on results of OGTT and other measures of glycemia

If an OGTT test result falls into the diabetes range, 14% (3/22) of Endocrinologists usually initiate treatment based on one abnormal test (**Table 3**). All (N=22) reported using insulin as one of the treatment modalities for CFRD (**Table 4**), however only 27% (6/22) use insulin as the sole treatment approach. Forty-one percent of Endocrinologists surveyed (9/22) usually or sometimes start insulin on a patient with impaired glucose tolerance/prediabetes. The rationale for starting insulin in this scenario included low body mass index (BMI)/difficulty gaining weight, declining lung function, recurrent pulmonary exacerbations and/or infections, rising HbA1c, and glucose trends (including reactive hypoglycemia, or hyperglycemia during overnight feeds). The majority of Endocrinologists (14/22 = 64%) have started insulin based on test results other than an OGTT or HbA1c in the diabetes range (i.e., Glucometer readings or CGM). The major theme for starting insulin in this context was treatment of glucose elevations in free living conditions.

**Table 3:** Endocrinologists’ Practice Management Based on Results of OGTT and Other Measures of Glycemia.

Reported Patient Management For Abnormal Glycemia	Frequency Reported			
	**Always/usually**	**Sometimes**	**Rarely**	**Never**
Initiate Treatment After One Abnormal OGTT Result	14% (3/22)	32% (7/22)	23% (5/22)	32% (7/22)
Use Another Test Method to Confirm Abnormal OGTT Result Before Starting Treatment	77% (17/22)	9% (2/22)	5% (1/22)	9% (2/22)
Start Insulin for Impaired Glucose Tolerance / Prediabetes	5% (1/22)	36% (8/22)	41% (9/22)	18% (4/22)

**Table 4:** Endocrinologists' reported treatment approaches for Cystic Fibrosis Related Diabetes (CFRD).

Reported Treatment for CFRD	Frequency Reported
**Insulin Only**	27% (6/22)
**Insulin + Another Treatment Option(s)**	74% (16/22)
**Metformin**	18% (4/22)
**Diet Changes Only**	64% (14/22)
**GLP-1**	18% (4/22)
**Sulfonylurea**	9% (2/22)
**Other medication(s)**	14% (3/22)

For patients on G-tube feeds, 81% (17/21) of Endocrinologists have started insulin/treatment based solely on fingerstick and/or CGM glucose readings. The rationale cited was that the fingerstick and/or CGM glucose readings were high enough to confirm the diagnosis, and/or laboratory glucose measurements were too difficult to obtain.

Twenty-one Endocrinologists (84%) who took the survey responded that they have discontinued insulin on a patient with CFRD. Of these, 52% (11/21) indicated that the patient only needed insulin during the hospitalization. Other reasons included normal HbA1c or other measures of glycemia in the context of reassuring health metrics; 3 respondents reported glucose improvements since starting HEMT.

## 4 Discussion

To our knowledge, a qualitative survey of CF Centers and Endocrinologists has not been previously conducted in the US to better understand provider perspectives on CFRD screening and management. Unfortunately, the current guidelines for CFRD screening can be challenging to implement. For the OGTT, a minimum of 8 hours fasting is required, and patients are instructed to consume a standard glucose beverage (Glucola) at a dose of 1.75 g/kg dextrose (maximum 75 g) within 5 minutes. Patients may have difficulty tolerating the taste of the Glucola or may not be able to drink the entire amount within the allotted time. In some cases, consumption of Glucola is followed by emesis, in which case the test must be discontinued (7). In addition, at least 2 separate blood draws are required, with a 2-hour time period between draws, and the patient is expected to rest during this interval. Many outpatient labs require individual venipuncture for each blood draw. Furthermore, if only one OGTT result is in the diabetes range, asymptomatic patients are recommended to have follow up testing performed to confirm a diagnosis of CFRD (4, 23). However, screening for CFRD is essential to the health of PwCF. CFF Patient Registry data show that CF centers screening more proactively for CFRD diagnose it earlier, and patients followed at CF centers with lower screening rates have greater decline in lung function by the time they are diagnosed with CFRD (5, 24). A Quality Improvement (QI) project for improving adherence to CFRD screening guidelines by the Mountain West Cystic Fibrosis Consortium (MWCFC) found that participating centers had low baseline screening adherence of 26.5%, which did not improve significantly throughout the 1-year study. However, the diagnosis rate improved from 12% to 17%, with improved adherence to CFRD management guidelines. In addition, at participating centers, patients had an associated improvement in their nutritional status (25).

However, there is currently minimal literature to address which portions of the OGTT testing process represent the greatest barriers to successful screening and are driving the current and persistently low rates of CFRD screening in the US (6). Our data found that both CF centers and Endocrinologists perceived significant barriers to completing recommend OGTT screening. Interestingly, although CF centers and Endocrinologists had differences in reported barriers, both groups selected test duration as the most significant barrier to OGTT completion. We also found substantial variations between the current CFF CFRD guidelines and reported practice at CF centers and by CF endocrinologists, including that some centers frequently obtain additional glucose time points during OGTTs, and many also utilize alternate diabetes screening methods, which may be in addition to or in place of the OGTT.

From our survey, the only reported process that was significantly associated with OGTT rates ≥50% across all respondent centers was the regular use of patient reminders regarding OGTT testing. Although pediatric centers had the highest ≥50% OGTT completion rates in the preceding year, only 60% reported ≥75% completion rates. It is unclear what is driving the higher OGTT completion rates in pediatric centers, as the use of reminders among pediatric centers was not statistically higher than adult or combined centers. It is possible that caregivers have a higher level of motivation to ensure their dependents with CF complete OGTT screening, although this was not specifically asked in our survey. Additionally, however, free commentary feedback from our study indicates that strategies some providers have effectively employed include long-term QI projects to help streamline the process and engage CF team members, early and ongoing education of patients and families regarding specific CF health outcomes related to CFRD and need for proactive screening, and flexibility in OGTT location/timing options to help address individual patient barriers. Asking about individual patients’ barriers to OGTT screening may help with devising individualized solutions.

Limitations to our study included utilization of an unvalidated survey and a suboptimal response rate (26). However, in creating the survey, questions were reviewed and revised by six different providers including endocrinologists and a pulmonologist – with the goal of minimizing leading questions and allowing opportunities for qualitative data capture with free-text comments from respondents. The survey was emailed once with a single reminder to the CFF center email distribution list. Based on correspondence with the CFF, the typical rate of opened emails is 20-30%. Therefore, our survey response rate of 18% of all CF centers represents a 60% response rate assuming a 30% email open rate. As with any survey, sampling bias exists. Despite a suboptimal survey response rate and not knowing the specific OGTT completion rates and characteristics of CF centers that did not respond to our survey, the health insurance demographics and OGTT completion rates of the CF centers that responded are overall similar to those reported in the CF registry (6). Moreover, the majority of pwCF receive care from a care center that is accredited by the CFF and/or affiliated with an academic institution (27). Our sample is therefore likely to be reasonably representative. The response rate among the endocrinologists surveyed was much better. This was likely at least partially because the endocrinologists were invited from individuals participating in the CFF sponsored EnVision program, who are specifically interested in CF and CFRD. Responses from these individuals would be expected to be more homogenous compared to responses from endocrinologists surveyed outside of this program. And yet, even amongst these providers with a declared career focused on CF-endocrinopathies, notable practice variation exists.

Current ISPAD CFRD guidelines are similar to CFF/American Diabetes Association CFRD screening guidelines (3, 4). Our findings are consistent with recent Canadian and French provider survey studies, which demonstrate provider deviation from CFRD screening guidelines, i.e., widespread use of HbA1c and CGM for screening, as well as utilizing non-insulin therapies for CFRD (10, 11). Italian CFRD Screening guidelines advocate for consideration of insulin therapy for pwCF with prediabetes and indeterminate glycemia if other health metrics indicate clinical instability (28). A growing body of literature in non-CF populations and more recent studies in pwCF have associated rises in earlier OGTT glucoses, including the 1 hour glucose, with beta-cell dysfunction and increased risk for development of diabetes (29–31). Our provider study further highlights the need for adequate, prospective studies, particularly in this HEMT era (32), to provide evidence-based data to update CFRD screening and management guidelines.

## 5 Conclusion and future directions

Although OGTT is considered the gold standard screening method for CFRD, completion rates remain suboptimal, and many providers are utilizing alternate glycemia screening methods. Having a reminder system for scheduling OGTTs may improve completion rates. Future studies are warranted to ascertain patient perceptions and barriers to diabetes screening and to determine what approaches align with management recommendations by experts but are also acceptable and feasible. Studies are also needed to further elucidate why pediatric CF centers have higher OGTT completion rates than adult CF centers, and whether there are modifiable factors that would change this outcome. Practice variation exists related to management of abnormal glycemia screening. Studies to improve our approach to CFRD screening, diagnostic cutoffs specific to the pwCF population, and treatment are urgently needed.

## Data availability statement

The raw data supporting the conclusions of this article will be made available by the authors, without undue reservation.

## Ethics statement

The studies involving human participants were reviewed and approved by University of California, Los Angeles Institutional Review Board. The patients/participants provided their written informed consent to participate in this study.

## Author contributions

First authorship: RH has first authorship. Contributions include conceptualization, writing – original draft of manuscript & editing, visualization. Senior authorship: CC has senior authorship. Contributions include conceptualization, writing – review & editing, visualization, supervision. Authorship: KO contributions include conceptualization, writing – review & editing, visualization, supervision. TV contributions include conceptualization and writing – review & editing. All authors contributed to the article and approved the submitted version.
