# Supplementary material for: A provider survey of cystic fibrosis related diabetes screening and management practices at North American CF centers
Source: Front Endocrinol (Lausanne). 2023 May 18;14:1183288. doi: 10.3389/fendo.2023.1183288 (PMC10232971; doi:10.3389/fendo.2023.1183288)
Supplement: Supplementary file 1 [file Table_1.docx]

**CFRD Screening Provider Surveys**

**2. Endocrinologists**

1. **CF Center Directors, Coordinators, or Designated Individual**

| Background Information:   1. Please indicate the name of your CF center: 2. Please identify your role in the CF center:   □ CF Center Director  □ Assistant/Associate CF Center Director  □ CF Pulmonologist (MD/DO/MBBS/NP/Advanced Practice Nurse/PA)  □ CF Center Coordinator  □ Other (please specify in comment)  □ Endocrinologist (note: survey ends and respondent is redirected to other survey)   1. In what type of setting is your CF center located?   □ Center affiliated with an academic institution  □ Community-based clinic/center that does not have an academic affiliation  □ Other (please specify in comment)   1. What percent of your CF patients have state-funded health insurance (i.e., Medicaid, etc.)?   □ <25%  □ 25 - <50%  □ 50 - <75%  □ ≥75%  □ I don’t know   1. What patient group does your CF center take care of?   □ Adult  □ Pediatric  □ Both adult and pediatric patients   1. What percent of eligible CF patients >= 10 years old at your center were screened for CFRD with an OGTT in the past year?   □ <25%  □ 25 - <50%  □ 50 - <75%  □ ≥75%  □ I don’t know   1. Does your center have an identified Endocrinologist(s) to whom you refer your CF patients?   □ Yes  □ No  □ Other (please specify in comment)   1. Does your center have an identified Endocrinologist who sees patients in a coordinated clinic with other CF providers?   □ Yes  □ No  Process for Ordering OGTT for CF Patients:  9. Do you have a protocol or standardized method for how OGTTs are ordered at your center?  □ Yes, we have a formal protocol  □ Yes, we have a standardized method, but no formal protocol  □ We are in the process of developing a standardized process  □ No, there is no standard process  -If yes, proceed to questions #10-16  -If no, skip to question #17   1. Who typically places the orders (even if a co-signer is needed) for OGTTs at your center? (please select all that apply):   □ CF Pulmonologist (MD/DO/MBBS/NP/Advanced Practice Nurse/PA)  □ CF Center Coordinator  □ RN or other nurse (not CF Center Coordinator)  □ Dietitian or other nutrition professional  □ Endocrinologist  □ Other (specify in comment)   1. Who typically contacts patients to schedule the OGTT? (please select all that apply):   □ Scheduler  □ CF Pulmonologist (MD/DO/MBBS/NP/Advanced Practice Nurse/PA)  □ CF Center Coordinator  □ RN or other nurse (not CF Center Coordinator)  □ Medical Assistant  □ Dietitian or other nutrition professional  □ Endocrinologist  □ Other (specify in comment)   1. Who typically provides directions to patients regarding how to prepare for the OGTT? (please select all that apply):   □ Scheduler  □ CF Pulmonologist (MD/DO/MBBS/NP/Advanced Practice Nurse/PA)  □ CF Center Coordinator  □ RN or other nurse (not CF Center Coordinator)  □ Medical Assistant  □ Dietitian or other nutrition professional  □ Endocrinologist  □ Other (specify in comment)   1. In what setting(s) do your patients have OGTTs done (please select all that apply)?:   □ Hospital-based lab affiliated with our center  □ Infusion center affiliated with our center  □ In our CF clinic  □ Outside lab  □ Other (specify in comment)   1. How is the glucose result obtained for the OGTT? (please select all that apply)   □ Insert IV and draw from it for each time point  □ Separate blood draw for each time point  □ Fingerstick glucose levels  □ Continuous glucose monitoring (CGM)   1. How often does your center provide routine reminders to patients when an OGTT is upcoming, due, and/or overdue?   □ Yes, always or almost always  □ Yes, sometimes  □ No  -If never, skip to question #17   1. What method is used for reminding patients to have their OGTT done (please select all that apply):   □ Automated Phone call  □ Phone call by live person  □ Communication through electronic medical record system  □ Email  □ Regular mail  □ Other (please specify in comment)  Glucose Source and Time Points for OGTT:   1. How often does your center use alternative glucose sources for OGTT testing (i.e., juice, pop/soda, jelly beans, licorice, etc.)?   □ Never  □ Rarely  □ Sometimes  □ Usually  □ Always  □ I don’t know   1. What OGTT time points for glucose levels does your center collect?   □ 0 and 120 min  □ 0, 60, and 120 min  □ 0, 30, 60, 90, and 120 min  □ Other time point(s) (specify in comment)  □ Time point(s) vary (per provider)  Other Diabetes Screening Methods:   1. Has your center used any of the following tests to screen/diagnose CFRD in the outpatient setting? (please select all that apply)   □ HbA1c  □ Fasting glucose (laboratory measurement)  □ Random glucose level (laboratory measurement)  □ Glucose level 2 hr after a meal (laboratory measurement)  □ Home fingerstick glucose monitoring  □ Continuous glucose monitoring (CGM)  □ Other (specify in comment)  □ None of the above  Diabetes Screening in Patients on G-tube feeds:   1. In CF patients >= 10 years old starting gastrostomy -tube feeds (bolus, nocturnal, or continuous), does your center recommend glucose monitoring during or after the feedings, to screen for diabetes?   □ Never  □ Rarely  □ Sometimes  □ Usually  □ Always  Barriers to OGTT Completion:   1. Please rank the top 5 perceived barriers to getting OGTTs done, in order from 1 (greatest barrier) to 5 (smallest barrier):   □ Number of blood draws  □ Duration of test  □ Uncertainty regarding how to order the test  □ Difficulty ensuring Glucola is available for the OGTT  □ Need for fasting before the test  □ Need for testing to be performed in the morning  □ Patient doesn’t like the taste of or doesn’t tolerate Glucola  □ Patient doesn’t think OGTT is important/necessary  □ Uncertainty regarding what to do with the results  □ I don’t know  □ Other (comment)  Additional Comments (Optional):   1. Please state what approaches your center has used to overcome barriers to OGTT screening (comment). 2. Please provide any additional comment(s) regarding diabetes screening in CF and/or feedback on this survey. 3. If willing, please email the link to the information sheet for a follow up Endocrinologist survey to Endocrinologist(s) affiliated with your CF center. | Background Information:   1. Please indicate the name of your affiliated CF center(s): 2. Please identify your background: (dropdown options)   □ Adult Endocrinologist  □ Pediatric Endocrinologist  □ Med-Peds Endocrinologist  □ Fellow in Endocrinology (Adult, Peds, or Med-Peds)  □ Other provider responsible for diabetes care   1. What age group of CF patients do you take care of? (please select all that apply)   □ 0-18 yr  □ 18-21 yr  □ 21-25 yr  □ >25 yr   1. In what outpatient setting do you care for patients with CF? (please select all that apply)   □ Patients are referred to my Endocrine and/or Diabetes clinic  □ I see patients in a coordinated clinic with a CF center team  □ Other (please specify in comment)   1. Which of the following best describes your endocrinology practice?   □ I see patients in a clinic/center affiliated with an academic institution  □ I see patients in a community-based clinic/center that does not have an academic affiliation  □ Other (please specify in comment)   1. Does your affiliated CF center(s) use OGTTs to screen for CFRD?   □ Yes  □ No   1. Do you personally order OGTTs to screen for CFRD at your CF center?   □ Yes  □ No  Location of OGTT for CF Patients:   1. In what setting(s) do your patients have OGTTs done? (please select all that apply):   □ Hospital-based lab affiliated with our center  □ Infusion center affiliated with our center  □ In our CF clinic  □ Outside lab  □ Other (specify in comment)  Glucose Source and Time Points for OGTT:  9. How often have you recommended using an alternative glucose source for OGTT testing (i.e., juice, pop/soda, jelly beans, licorice, etc.)?  □ Never  □ Rarely  □ Sometimes  □ Usually  □ Always  □ Other (specify in comment)   1. What OGTT glucose time points are collected?   □ 0 and 120 min  □ 0, 60, and 120 min  □ 0, 30, 60, 90, and 120 min  □ Other time point(s) (specify in comment)  □ Time point(s) vary  Other Diabetes Screening Methods:   1. Have you used any of the following tests to screen/diagnose CFRD in the outpatient setting? (please select all that apply)   □ HbA1c  □ Fasting glucose (laboratory measurement)  □ Random glucose (laboratory measurement)  □ Glucose 2 hr after a meal (laboratory measurement)  □ Home fingerstick glucose monitoring  □ Continuous glucose monitoring (CGM)  □ Other (specify in comment)  OGTT Results and Interventions:   1. If an OGTT test result falls into the diabetes range, do you initiate treatment based on that one test?   □ Never  □ Rarely  □ Sometimes  □ Usually  □ Always  □ Comments (optional)   1. How often do you use another test or method to confirm glucose abnormalities or diabetes, prior to initiating treatment?   □ Never  □ Rarely  □ Sometimes  □ Usually  □ Always  □ Comments (optional)   1. What treatments have you used for CFRD? (please select all that apply)   □ Insulin  □ Metformin  □ Sulfonylurea  □ GLP-1 agonist  □ Diet changes only  □ Other medication(s)   1. How often have you started insulin in a patient with impaired glucose tolerance/prediabetes?   □ Never  □ Rarely  □ Sometimes  □ Usually  □ Always  □ Comments (optional)  -If never, skip to question #17   1. If you have started insulin/treatment in a patient with impaired glucose tolerance/prediabetes, what factors influenced this decision? (please select all that apply)   □ Low weight/BMI  □ Low/worsening PFTs  □ Glucose/HbA1c level(s) trending upward  □ Recurrent pulmonary exacerbations and/or infections  □ Other (please specify)   1. In the outpatient setting, have you started insulin based on test results other than an OGTT or HbA1c in the diabetes range (i.e., Glucometer readings, CGM)?   □ Yes  □ No   1. In the outpatient setting, have you ever discontinued insulin in a patient with CFRD previously on insulin treatment?   □ Yes – patient only required insulin during  hospitalization  □ Yes – other (specify in comment)  □ No   1. For patients on G-tube feeds, have you started insulin/treatment based solely on fingerstick and/or CGM glucose readings?   □ Yes  □ No   1. If you have started insulin/treatment in a patient on G-tube feeds based solely on fingerstick and/or CGM glucose level(s), please select all reason(s) that apply:   □ Unable to obtain laboratory glucose measurement in setting of home overnight feeds  □ Patient refused/did not do blood draw  □ Glucose levels via fingersticks and/or CGM were high enough to confirm CFRD  □ Other (please specify)  Barriers to OGTT Completion:   1. Please rank the top 5 perceived barriers to getting OGTTs done, in order from 1 (greatest barrier) to 5 (smallest barrier):   □ Number of blood draws  □ Duration of test  □ Uncertainty regarding how to order the test  □ Difficulty ensuring Glucola is available for the OGTT  □ Need for fasting before the test  □ Need for testing to be performed in the morning  □ Patient doesn’t like the taste of or doesn’t tolerate Glucola  □ Patient doesn’t think OGTT is important/necessary  □ Uncertainty regarding what to do with the results  □ Other (comment)  Additional Comments:   1. Please state what approaches you have you used to overcoming barriers to OGTT screening (comment). 2. Please indicate any additional comment(s) regarding diabetes screening in CF patients and/or this survey: 3. If willing, please email the information sheet to this survey to an Endocrinologist(s) affiliated with your CF center so this survey may be sent to them as well. |
| --- | --- |
